# Community evaluation of VECTRON™ T500, a broflanilide insecticide, for indoor residual spraying for malaria vector control in central Benin; a two arm non-inferiority cluster randomised trial

**DOI:** 10.1038/s41598-023-45047-w

**Published:** 2023-10-19

**Authors:** Corine Ngufor, Renaud Govoetchan, Augustin Fongnikin, Corneille Hueha, Juniace Ahoga, Thomas Syme, Abel Agbevo, Abdoulaye Daleb, Graham Small, Derric Nimmo, John Bradley, Rock Aikpon, Laurent Iyikirenga, Razaki Osse, Filemon Tokponnon, Germain Gil Padonou

**Affiliations:** 1 London School of Hygiene and Tropical Medicine (LSHTM), London, WC1E 7HT UK; 2 Centre de Recherches Entomologiques de Cotonou (CREC), Cotonou, Benin; 3 Panafrican Malaria Vector Research Consortium (PAMVERC), Cotonou, Benin; 4 National Malaria Control Programme, Ministry of Health, Cotonou, Benin; 5 Innovative Vector Control Consortium, Liverpool, UK; 6 PMI VectorLink, ABT Associates, Cotonou, Benin

**Keywords:** Ecology, Zoology

## Abstract

VECTRON™ T500 is a wettable powder IRS formulation of broflanilide, a newly discovered insecticide. We performed a two-arm non-inferiority community randomised evaluation of VECTRON™ T500, compared to Fludora® Fusion against pyrethroid-resistant *Anopheles gambiae* s.l. in an area of high coverage with pyrethroid-only nets in the Za-Kpota District of central Benin. One round of IRS was applied in all consenting households in the study area. Sixteen clusters were randomised (1:1) to receive VECTRON™ T500 (100 mg/m^2^ for broflanilide) or Fludora® Fusion (200 mg/m^2^ for clothianidin and 25 mg/m^2^ for deltamethrin). Surveys were performed to assess adverse events and the operational feasibility and acceptability of VECTRON™ T500 among spray operators and household inhabitants. Human landing catches were conducted in 6 households every 1–2 months for up to 18 months post-intervention to assess the impact on vector densities, sporozoite rates and entomological inoculation rates. Bottle bioassays were performed to monitor vector susceptibility to pyrethroids, broflanilide and clothianidin. Monthly wall cone bioassays were conducted for 24 months to assess the residual efficacy of the IRS formulations using susceptible and pyrethroid-resistant *An. gambiae* s.l. A total of 26,562 female mosquitoes were collected during the study, of which 40% were *An. gambiae* s.l*.*, the main malaria vector in the study area. The vector population showed high intensity pyrethroid resistance but was susceptible to broflanilide (6 µg/bottle) and clothianidin (90 µg/bottle). Using a non-inferiority margin of 50%, vector density indicated by the human biting rate (bites/person/night) was non-inferior in the VECTRON™ T500 arm compared to the Fludora® Fusion arm both indoors (0.846 bites/p/n in Fludora® Fusion arm vs. 0.741 bites/p/n in VECTRON™ T500 arm, IRR 0.54, 95% CI 0.22–1.35, p = 0.150) and outdoors (0.691 bites/p/n in Fludora® Fusion arm vs. 0.590 bites/p/n in VECTRON™ T500 clusters, IRR 0.75, 95% CI 0.41–1.38, p = 0.297). Sporozoite rates and entomological inoculation rates did not differ significantly between study arms (sporozoite rate: 0.9% *vs* 1.1%, p = 0. 0.746, EIR: 0.008 vs 0.006 infective bites per person per night, p = 0.589). Cone bioassay mortality with both VECTRON™ T500 and Fludora® Fusion was 100% for 24 months post-IRS application on both cement and mud treated house walls with both susceptible and pyrethroid-resistant strains of *An. gambiae* s.l. Perceived adverse events reported by spray operators and householders were generally very low (< 6%) in both study arms. VECTRON™ T500 was non-inferior to Fludora® Fusion in reducing the risk of malaria transmission by pyrethroid resistant vectors when applied for IRS in communities in central Benin. The insecticide showed prolonged residual efficacy on house walls, lasting over 24 months and had a high acceptability with homeowners. Community application of VECTRON™ T500 for IRS provides improved and prolonged control of pyrethroid resistant malaria vectors and enhances our capacity to manage insecticide resistance.

## Introduction

Indoor residual spraying (IRS) is a core intervention for the prevention and control of malaria. It significantly reduces adult mosquito vector densities, thereby disrupting malaria transmission for several months following its application. The largescale implementation of IRS contributed substantially to the elimination of malaria from 37 countries during the Global Malaria Elimination Campaign (1955–1969)^1^. Following this success, IRS was expanded to malaria endemic countries in the African continent resulting in effective malaria control in diverse endemic settings over the past decades^2–6^. Modelling has indicated that the sustained deployment of IRS in high endemic settings was a major contributing factor to the substantial reductions in malaria burden observed between 2000 and 2015^7^.

Despite its impact, the use of IRS has, unfortunately, declined in malaria endemic African countries in recent years mostly due to concerns about the cost and logistical challenges associated with its implementation^8,9^. IRS programmes funded by the US President’s Malaria Initiative (PMI) have been withdrawn from several African countries, largely attributed to the higher costs required to sustain annual IRS campaigns using more expensive non-pyrethroid insecticide formulations^8^. This withdrawal has seen increasing reports of rebounds in malaria transmission and number of malaria cases in recent years^10–12^ demonstrating that the declining use of IRS may impact upon progress against malaria and achieving elimination targets.

The development and spread of vector resistance to the rather limited number of insecticides that have been used for malaria vector control is well documented^13^. The recent stall in progress in malaria control has been partly attributed to widespread insecticide resistance in local malaria vector populations, resulting in sub-optimal levels of malaria control^14^. One strategy proposed for managing insecticide resistance in malaria vectors and improving vector control is the rotational use of different insecticide classes for IRS^15^. This will, however, require the development of a more diverse portfolio of IRS insecticides presenting different modes of action. This is driving the search for new IRS insecticide chemistries that can provide prolonged control of insecticide-resistant malaria vector populations and contribute to insecticide resistance management via rotations.

VECTRON™ T500 is a wettable powder IRS formulation of the meta-diamide broflanilide, a new insecticide developed by Mitsui Chemicals Crop & Life Solutions, Inc^16^. Broflanilide acts as a non-competitive antagonist of the γ-aminobutyric acid (GABA) receptor of chloride channels of the insect inhibitory nervous system thus presenting a new mode of action for malaria vector control^17^. Laboratory and semi-field experimental hut studies conducted in Benin, Burkina Faso and Tanzania have shown the potential of VECTRON™ T500 to provide improved and extended control of pyrethroid-resistant *Anopheles gambiae* s.l. and *An. arabiensis* compared to other WHO prequalified IRS products^18–20^. Based on these findings, VECTRON™ T500 was recently added to the WHO list of prequalified IRS products^21^ thus becoming available for procurement by malaria control programmes. Laboratory bioassays have also indicated the absence of cross-resistance to broflanilide and other insecticide resistance mechanisms prevalent in malaria vectors in Africa, demonstrating its suitability for large-scale deployment in most endemic countries^18,22,23^.

More recently, community trials have been conducted across Africa to assess the impact of deploying IRS with VECTRON™ T500 on a large-scale for malaria vector control in endemic communities. Here we present results of a large-scale cluster randomised trial that evaluated the entomological efficacy, residual activity, and acceptability of VECTRON™ T500 for indoor residual spraying against pyrethroid-resistant malaria vectors in communities in central Benin, in comparison to Fludora® Fusion, a WHO prequalified IRS clothianidin-based formulation used in previous campaigns in Benin. The trial ran for a total of 24 months in the Za-Kpota District of Benin.

## Methods

### Study arms

The following IRS arms were compared in the study:

- VECTRON™ T500 is a novel wettable powder IRS insecticide formulation of broflanilide (CAS 1207727-04-5), a meta-diamide insecticide. It was discovered and manufactured by Mitsui Chemicals Crop & Life Solutions, Inc. VECTRON™ T500 was supplied in 50 g sachets and applied at a target rate of 100mg of active ingredient per m^2^ for broflanilide. VECTRON™ T500 was added to the WHO PQT/VCP list in March 2023.
- Fludora® Fusion is a WHO PQT/VCP listed wettable powder IRS formulation of a mixture of clothianidin (a neonicotinoid) (CAS 210880-92-5): 50% and deltamethrin (CAS 52918-63-5): 6.25%. It is manufactured by Bayer S.A.S./ENVU and was supplied in sealed water-soluble bags packaged in 100 g sachets. Fludora® Fusion served as the reference product against which the efficacy of VECTRON™ T500 was assessed. The insecticide was applied at a target rate of 200 mg of active ingredient per m^2^ for clothianidin and 25 mg of active ingredient per m^2^ for deltamethrin.

### Study area

The study was performed in the 15 villages of the Za-Kpota centre sub-District situated in the Za-Kpota District (7.2169° N, 2.1843° E) of the Zou Department of Benin. Malaria is highly endemic in the district, with a peak during the rainy season which runs from May to October. A baseline census was conducted in the study area in 2020 to collect data on the distribution, location, number, and accessibility of households as well as the type and use of available ITNs. Overall, 37,007 people were found living in 9080 households consisting of a total of 12,608 structures. Approximately 91% of households owned insecticide-treated nets (ITNs), which were all treated only with pyrethroids. The most common ITN brand was PermaNet® 2.0, accounting for 80% of all ITNs found in the study area. The main malaria vector in the study area is *An. gambiae* s.l.*,* consisting of a combination of *An. coluzzii* and *An. gambiae* s.s. The vector population exhibits intense resistance to pyrethroids but is largely susceptible to organophosphates and carbamates.

### Study design, sample size and randomisation

**Figure 1:**
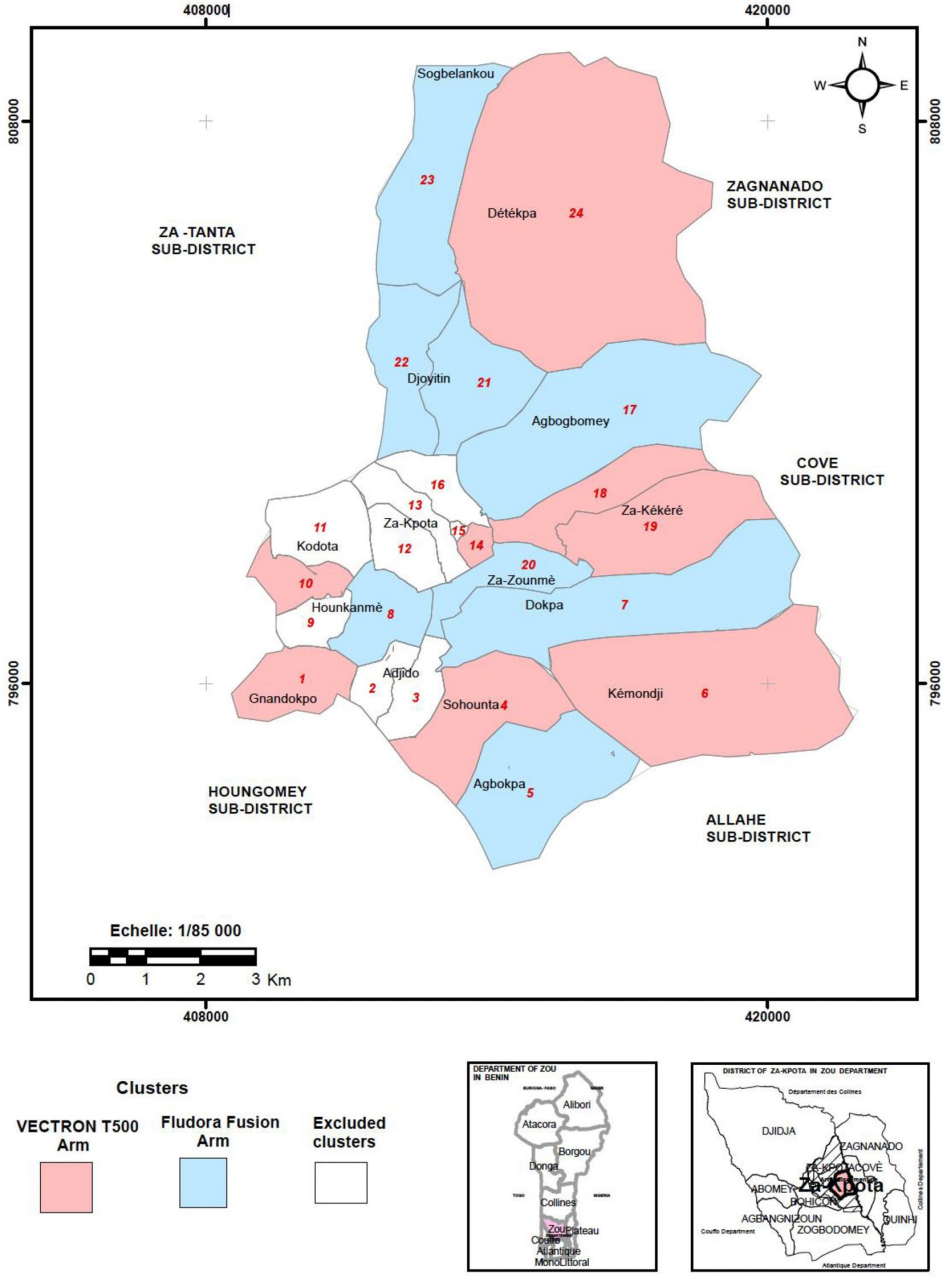
Spatial distribution of IRS study clusters in VECTRON™ T500 (pink) and Fludora® Fusion (light blue) arms in the Za-Kpota sub-District of Benin. Clusters in white were treated but are not included in the post-IRS evaluation studies.

The study was designed as a non-inferiority trial to determine if VECTRON™ T500 was no worse than Fludora® Fusion in terms of its impact on the densities of the main malaria mosquito vector in the study area. Power analysis showed that, using 8 clusters per arm and at least 49-man night collections per cluster, the trial had 80% power to show that VECTRON™ T500 would not lead to higher mean numbers of mosquitoes per man night assumed in the Fludora® Fusion arm by a non-inferiority margin of 50%. The baseline census data was used to divide the study area into 24 clusters, each consisting of one village or a group of village hamlets with approximately 150–500 households. The individual clusters were the unit of randomisation. Eight of these clusters were eliminated due to inaccessibility, poor acceptance of the study and low vector density as revealed by the baseline entomological surveys. The remaining 16 study clusters were pair matched based on baseline *An. gambiae* s.l. densities and one member of each pair was randomly allocated to each study arm (VECTRON™ T500 or Fludora® Fusion) (Fig. 1). The randomisation was repeated multiple times to ensure that the study arms were also similar in terms of population size, number of households and ITN ownership. Each study cluster consisted of a core area and a buffer zone. Core areas of clusters were 0.5 km apart to reduce infiltration of mosquitoes from other clusters. The study was single-blinded; the inhabitants of each cluster were masked as to the type of IRS product they received as were the mosquito collectors. The project delivered the IRS intervention to all households in all 15 administrative villages within the sub-district, including the clusters that were excluded from the evaluation.

Following IRS application, studies were conducted to assess the perceived adverse events and acceptability of each IRS product by homeowners and the entomological impact of each intervention on the vector populations was assessed with regards to vector species composition, vector density, resting behaviour, insecticide susceptibility, human biting rate, sporozoite rate and entomological inoculation rate. WHO cone bioassays were also performed at monthly intervals to investigate the residual efficacy of each IRS formulation.

### Entomological surveys

Three rounds of entomological surveys were conducted before IRS application (between October 2020 to April 2021). In the period post IRS application, the first mosquito collections were performed 1 month after and subsequently every 2 months for up to 18 months. During each round, data were collected on the abundance, biting behaviour, and sporozoite rate of the main malaria vector in each village/cluster in the study area. Mosquito sampling was primarily performed using human landing catches (HLCs). During each collection round, HLCs were performed for 2 nights, in randomly selected houses in each cluster. Six houses per cluster were used per HLC round per cluster both pre and post-IRS intervention. For each house, 4 consenting human volunteer mosquito collectors worked in pairs with one collecting mosquitoes indoors and the other outdoors. Collections were performed between 07:00 pm and 07:00 a.m. with pairs alternating after 6 h i.e., 07:00 p.m. to 01:00 a.m. and 01:00 a.m. to 07:00 a.m. Collectors sat on chairs indoors or outdoors with their legs exposed and used flashlights, catching all mosquitoes landing on their legs with haemolysis tubes. To allow assessment of peak mosquito biting hours, each hour’s collection was kept separately.

Mosquitoes sampled from each household were counted and morphologically identified to species using appropriate taxonomic keys^24^ and further analysed for *Plasmodium* sporozoites using CSP-ELISA and for sibling species identification using the SINE PCR technique. The following outcome measures were estimated for each cluster:

- Vector density indicated by the number of main vector mosquitoes collected by HLC per night/house
- Human biting rate (HBR) indicated by the number of main vector mosquitoes collected through HLC per number of man nights.
- Sporozoite rate (SR) indicated by the proportion of sporozoite positive main vector mosquitoes identified through CSP-ELISA.
- Entomological Inoculation Rate (EIR) estimated by HBR x SR.

To investigate the mechanisms of resistance in the vector population and how these may have changed post-intervention, a sub-sample of wild adult mosquitoes collected in households were analysed for the presence of target site insecticide resistance genes. Detection of L1014F *Kdr* and G119S *Ace-1* mutations were performed following the protocols described by Martinez-Torres et al. (1998)^25^ and Weill et al. (2004)^26^ respectively.

### Insecticide susceptibility monitoring

The susceptibility of the vector population in the study area to relevant insecticides was assessed at baseline and at 3- and 10-months post IRS intervention. Mosquitoes for susceptibility bioassays were sampled as larvae from breeding sites in selected clusters within the study area and raised to F1 in the insectary before testing. Villages/clusters were selected based on their accessibility and on availability of mosquito larval breeding sites. Bottle bioassays were used to assess the susceptibility of mosquitoes from each village to clothianidin and broflanilide. Clothianidin was tested at a discriminating dose of 90 µg/bottle as specified by the manufacturer while broflanilide was tested at 6 µg/bottle as determined in preliminary dose–response bioassays^22^. To prevent crystallization of the insecticide in bottle bioassays and to improve bioavailability and uptake by exposed mosquitoes, Mero® (81% rapeseed oil methyl ester) was used as an adjuvant at a dose of 800 ppm for broflanilide and 1000 ppm for clothianidin. The intensity of resistance to deltamethrin in each study arm was also assessed at baseline and at 3- and 10-months post IRS in bottle bioassays with bottles treated at 1 × and 10 × the diagnostic dose of 12.5 µg/bottle. Knockdown was recorded after 1 h and mortality was recorded after 24 h for deltamethrin, after 72 h for broflanilide and after 120 h for clothianidin. At each time point, a total of 300–400 unfed 3- to 5-day-old, field collected *An. gambiae* s.l. mosquitoes from each study arm were exposed to each insecticide treatment in replicates of 20–25 mosquitoes per bottle.

### Assessment of perceived adverse events and acceptability

To identify and document any perceived adverse events associated with the IRS application of each insecticide, a questionnaire was administered to each spray operator after each day of spraying, at the end of the IRS campaign and one-month post-spraying. A questionnaire was also administered to the heads of households of randomly selected households in each study arm immediately after spraying and was repeated at 6 months post-spraying. Study participants were encouraged to contact members of the study team if they experienced health issues that they believed were related to the IRS applications.

### Residual activity of IRS insecticides on house walls

To assess insecticide bioavailability and residual activity on sprayed walls, monthly WHO cone bioassays were performed in four randomly selected households (2 with mud walls and 2 with cement walls) in the 8 evaluation clusters for each study arm. Forty (40) laboratory-maintained 2–5-day-old, unfed insecticide susceptible *An. gambiae* s.s. Kisumu or pyrethroid-resistant *An. gambiae* s.l. Covè strain female mosquitoes were exposed for 30 min in cohorts of 10 mosquitoes per cone and 1 cone per wall in the living room of each selected house.

- *An. gambiae* s.s. Kisumu strain is an insecticide-susceptible reference strain, which originated from Kisumu, western Kenya.
- *An. gambiae* s.l. Covè strain is highly resistant to pyrethroids and organochlorines but susceptible to other insecticide classes including broflanilide and clothianidin. Pyrethroid resistance is mediated by high frequencies of the *Kdr* L1014F mutation (> 80%) and overexpression of cytochrome P450 monooxygenases.

The WHO wall cone bioassays were performed every month for 12 months post IRS application and subsequently every two months up to 18 months and finally at 24 months post IRS application. The same houses were used throughout the study. After exposure, mosquitoes were held in netted plastic cups and provided with a 10% glucose solution via a piece of cotton wool. Immediate knockdown was recorded 1 h after exposure and delayed mortality was recorded every 24 h for up to 120 h. Cone location on the walls was marked at each time point and nearby locations were chosen for each subsequent round of cone bioassays. A control cone with ten mosquitoes was set up on an unsprayed plywood board outside of each sprayed house in a shaded area close to the house. Due to the limited availability of mosquitoes of the pyrethroid-resistant Covè strain, only 4 clusters per arm were tested during the monthly cone bioassays using this strain while all 8 study clusters per arm were tested with the susceptible *An. gambiae* s.s. Kisumu strain.

### IRS campaign, coverage, and quality

One round of indoor residual spraying was conducted in the study area before the start of the long rainy season and main malaria transmission season. The IRS campaign ran for 22 days between 15 May and 9 June 2021. It was organized by the project team in collaboration with trained experts from the National Malaria Control Programme (NMCP) and PMI VectorLink Project Benin. To ensure community participation, all consenting households in all 15 administrative villages within the entire sub-district were sprayed including clusters that were excluded from the evaluation. A total of 11,336 structures out of the 12,933 eligible structures (as defined by WHO operational guidelines ^27^) found in the 15 administrative villages, were sprayed by spray operators resulting in 88% spray coverage which is above the required target of 80%. The average coverage rate was 83% and 93% respectively in the VECTRON™ T500 and Fludora® Fusion arms. A total of 3889 sachets of insecticide were used of which 2124 sachets were VECTRON™ T500 and 1765 sachets were Fludora® Fusion. The actual total number of sachets used per cluster was also recorded and compared to the quantity of insecticide required based on calculations of spray coverage of eligible structures to provide a proxy estimate of IRS quality.

Before spraying, 4 filter papers (Whatman No. 1) measuring 5 cm × 5 cm were fixed on the 4 walls (1 per wall) of one room in 5 randomly selected households/cluster for each of the 8 evaluation clusters per study arm. After spraying, they were left to dry for 2–3 h, wrapped in aluminium foil, and stored at 4 °C (± 2 °C) after which they were shipped to the Liverpool School of Tropical Medicine for chemical analysis by HPLC using validated methods for extraction and analysis of broflanilide, clothianidin and deltamethrin.

### Ethical considerations

The study was approved by the Ethics Review Committees of Ministry of Health Benin (Ethical decision n°29 of 13 August 2020) and the London School of Hygiene and Tropical Medicine (Ref: 22453 of 18 August 2020) and all methods were performed in accordance with the relevant guidelines and regulations. Prior to any project activities, village and hamlet leaders were invited to sensitization sessions conducted by district health officers and written informed consent was sought from the local leader before starting data collection. Heads of households involved in the study gave informed consent prior to their participation in the study. The consent form was written in French and indicated the purpose of the study, the procedures, risks, and benefits. Participants were informed that participation was completely voluntary, and that they could withdraw from participation at any time. Where necessary, the consent form was explained to them in their local language by a trained interpreter. Participants were asked to sign the consent form in duplicate, one copy was kept by the project, and they retained the other copy. Where the individual was unable to read or write, their fingerprint was taken, and a signature was obtained from a witness to the informed consent procedure.

Informed consent was also obtained from volunteer mosquito collectors for HLCs. Only male individuals aged between 18 and 40 years old and who were members of the local community were recruited and trained as mosquito collectors. Collectors were free to withdraw from the trial at any time. As mosquito collectors were residents and had regularly been exposed to biting and malaria, they were not offered chemoprophylaxis. However, they were regularly examined by a study physician for clinical signs of malaria while participating in the study and referred to the nearest local health centre for free treatment if diagnosed positive for malaria. Mosquito collectors also had free access to malaria diagnosis and treatment up to 4 weeks after the end of the study. A study physician at the local public health facility was available to administer all medical care under the project. Spray operators were males, at least 18 years old, physically fit, and healthy, had no obvious disabilities that would limit their mobility and were literate enough to read and write French to be able to follow all insecticide handling procedures safely.

### Data management and statistical analysis

Household data collected during the census and surveys were captured on electronic forms using smartphones installed with OpenDataKit (ODK) Collect. Entomological data were collected on pre- designed data collection forms and then double-entered into pre-established Microsoft Excel databases. All data are stored on a secure server located at the CREC/LSHTM Facility in Benin. Count data on mosquito densities was analysed by comparing log transformed cluster level rates using linear regression. Terms for matched pair and baseline density were included. This method was used because other methods for clustered data, such as random effect models, do not perform well with only 8 clusters per arm^28^. Density rate ratios (incidence rate ratio) of the geometric mean of cluster level rates between the VECTRON™ T500 and Fludora® Fusion study clusters for human biting rates pre and post IRS application are presented. Proportional data on secondary outcomes related to sporozoite rates (proportion of plasmodium positive mosquitoes), indoor vs. outdoor biting and *An. gambiae* s.l. sibling species composition were analysed using linear regression on cluster level sporozoite rates, adjusted for matched pair of clusters. Entomological inoculation rate (sporozoite rate × HBR) was calculated at the cluster level for each timepoint and analysed the same way as sporozoite rates. All analysis were performed on Stata version 17.

In situ wall cone bioassay knockdown and mortality data was pooled across households of each substrate type for each arm and plotted against the number of months since spraying and residual efficacy of each IRS treatment was expressed as the number of months for which overall mortality for each mosquito species and each wall substrate type remained ≥ 80%.

## Results

### Baseline characteristics

From the baseline census and entomological surveys performed in October 2020, a total of 37,007 people were found living in 12,608 households in the study area (Za-Kpota central sub-district). A total of 16 clusters were randomised to the VECTRON™ T500 or Fludora® Fusion arms based on vector density (8 clusters per arm). Both arms were similar in terms of population size, number of households and ITN coverage and key entomological indicators at baseline (Table 1). ITNs found in the households in the study area were treated only with pyrethroids and the most prevalent brands were Yorkool® and PermaNet® 2.0 that had been distributed to householders by the NMCP in the 2020 mass campaign. The baseline human biting rate of the main vector (*An. gambiae* s.l.), did not differ significantly between the study arms either indoors (3.15 vs 2.52, p = 0.97) or outdoors (2.38 vs 2.52, p = 0.85).

**Table 1:** Baseline characteristics of clusters in each study arm.

Characteristics	VECTRON T500	Fludora fusion
N clusters	8	8
Population	12,393	11,278
N households	3030	3005
N structures	6943	5990
ITN usage (%)	93	91
Mean indoor human biting density (per person per night)	3.15	2.52
Mean outdoor human biting density (per person per night)	2.38	2.08
% *Anopheles gambiae* s.l. in mosquito population	40	43
Entomological inoculation rate (EIR)*	0.05	0.04
Intensity of resistance to pyrethroids	High	High
Susceptibility to broflanilide—6 µg/bottle (% mortality)	95	96
Susceptibility to clothianidin—90 µg/bottle (% mortality)	100	100

*Calculated as sporozoite rate × human biting rate.

### Mosquito species composition

**Figure 2:**
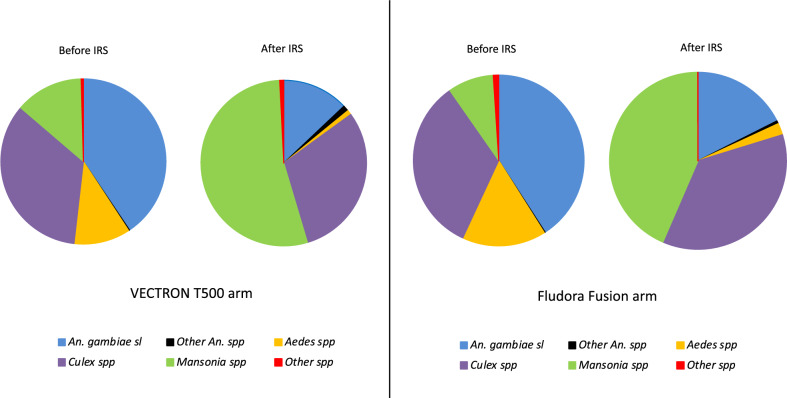
Mosquito species composition in VECTRON™ T500 and Fludora® Fusion arms before and after IRS intervention.

A total of 26,562 female mosquitoes were collected in the study area over the trial period using HLC. Overall mosquito species composition was generally similar between both study arms (Fig. 2). *An. gambiae* s.l. constituted the largest proportion at baseline (40% in the VECTRON™ T500 arm and 43% in the Fludora® Fusion arm), while the *Mansonia* spp. were the most prevalent after the IRS application (56% in the VECTRON™ T500 arm and 43% in the Fludora® Fusion arm) (Fig. 2).

**Figure 3:**
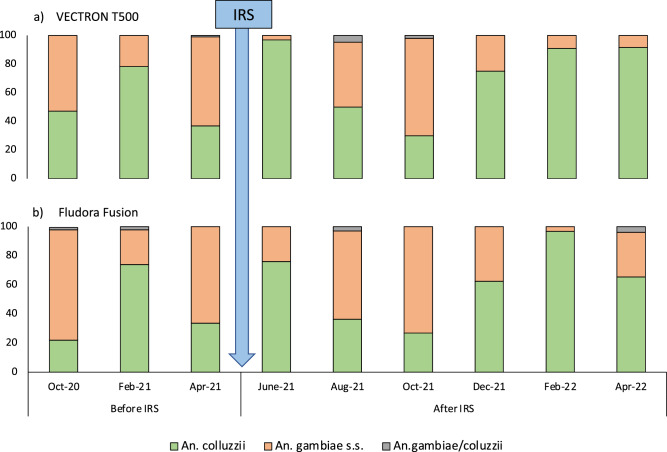
Variation in the species composition in *An. gambiae* complex collected by human landing collection in VECTRON™ T500 (**a**) and Fludora® Fusion (**b**) clusters before and after IRS intervention. Y-axis refers to % of each sibling species.

A total of 920 samples of *An. gambiae* s.l. from HLC collections before IRS application and up to 12 months after IRS, were analysed for sibling species identification following the protocol described by Santolamazza et al. (2008). *An. coluzzii* and *An. gambiae* s.s*.* were the 2 species identified. Before IRS, *An. gambiae* s.s. was the more abundant species representing 51% and 58% in VECTRON™ T500 and Fludora® Fusion arms respectively. After IRS, *An. gambiae* s.s. remained the predominant species in the Fludora® Fusion arm (58%), while *An. coluzzii* became more prevalent in the VECTRON™ T500 arm, constituting 68%. The trend in species composition per time point showed a seasonal fluctuation between the *An. coluzzii* and *An. gambiae* s.s. sibling species in both study arms (Fig. 3). The largest proportions of *An. coluzzii* were observed in the months of June 2021 just after the application of the IRS insecticides and in the drier months from December to April.

### Impact on vector density

A total of 5,397 *An. gambiae* s.l. vector mosquitoes were collected by HLC during the trial of which 3,005 were collected indoors and 2,392 outdoors. Mosquito densities were consistently higher indoors compared to outdoors in both study arms both before and after the IRS intervention (p < 0.05). Peak mosquito biting time was observed between 12:00 am and 6:00 am in both study arms before and after the IRS intervention.

Table 2 presents the numbers collected, human biting rates and rate ratios between the VECTRON™ T500 and Fludora® Fusion arms before and after intervention. At baseline, human biting rate (HBR) was similar between both study arms both indoors (2.521 bites per person per night (bites/p/n) in Fludora® Fusion clusters vs. 3.153 bites/p/n in VECTRON™ T500 clusters, IRR 0.98, 95% CI 0.51–1.86, p = 0.929) and outdoors (2.080 bites/p/n in Fludora® Fusion clusters vs. 2.382 bites/p/n in VECTRON™ T500 clusters, IRR 0.91, 95% CI 0.58–1.42, p = 0.624). These results confirm the baseline comparability of the study arms in terms of the primary outcome of vector density. After IRS application, the HBR declined in both study arms compared to the baseline period but did not differ significantly between the study arms both indoors (0.846 bites/p/n in Fludora® Fusion arm vs. 0.741 bites/p/n in VECTRON™ T500 arm, IRR 0.54, 95% CI 0.22–1.35, p = 0.150) and outdoors (0.691 bites/p/n in Fludora® Fusion arm vs. 0.590 bites/p/n in VECTRON™ T500 clusters, IRR 0.75, 95% CI 0.41–1.38, p = 0.297). A similar trend was observed when indoor and outdoor data were combined.

**Table 2:** Density of main malaria vector (*An gambiae* s.l.) in human landing collections before and after IRS intervention.

Phase	Study arm	Total collected	Person nights	HBR	Rate ratio* (95% CI)	P value
Indoor collections						
Before IRS	Fludora fusion	726	288	2.521	0.98 (0.51, 1.86)	0.929
	VECTRON T500	908	288	3.153		
After IRS	Fludora fusion	731	864	0.846	0.54 (0.22, 1.35)	0.150
	VECTRON T500	640	864	0.741		
Outdoor collections						
Before IRS	Fludora Fusion	599	288	2.08	0.91 (0.58, 1.42)	0.624
	VECTRON T500	686	288	2.382		
After IRS	Fludora Fusion	597	864	0.691	0.75 (0.41, 1.38)	0.297
	VECTRON T500	510	864	0.590		
Indoor and outdoor collections						
Before IRS	Fludora Fusion	1325	576	2.3	0.94 (0.54, 1.62)	0.786
	VECTRON T500	1594	576	2.767		
After IRS	Fludora Fusion	1328	1728	0.769	0.66 (0.32, 1.36)	0.213
	VECTRON T500	1150	1728	0.666		

*Ratio of geometric means of cluster level rates.

**Figure 4:**
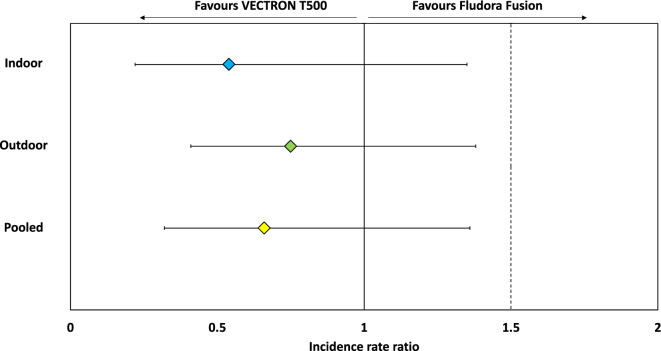
Non-inferiority assessment of vector density in VECTRON™ T500 arm compared to Fludora® Fusion arm post IRS intervention.

**Figure 5:**
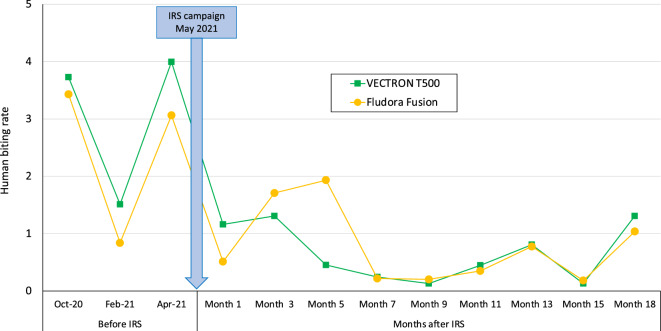
Human biting rate of *An. gambiae* s.l. in both VECTRON™ T500 and Fludora® Fusion arms before and after IRS implementation.

The upper 95% confidence intervals of the density rate ratios between VECTRON™ T500 and Fludora® Fusion were < 1.5 both indoors and outdoors and when combined (Fig. 4). This finding shows that, using the predefined non-inferiority margin of 50%, VECTRON™ T500 was no worse than Fludora® Fusion in reducing the densities of the main malaria vector (*An. gambiae* s.l.) either indoors or outdoors when deployed for indoor residual spraying in the study area. Figure 5 presents the change in HBR in both study arms over time. Apart from the collection round performed in month 5, combined *An. gambiae* s.l. biting rates were generally similar between both study arms across the collection rounds.

### Impact on sporozoite rate and EIR

Overall, 4,831 specimens of *An. gambiae* s.l. collected across the study area were analysed for *P. falciparum* detection by ELISA and the results are presented in Table 3. The sporozoite rate (SR), calculated as the % of positive mosquitoes divided by the total number tested, was similar between both study arms before IRS (1.9% with Fludora® Fusion and 2% with VECTRON™ T500, p = 0.692) showing that both arms were comparable at baseline. After IRS applications, the sporozoite rate reduced in both study arms compared to baseline (p < 0.001) and was lower with VECTRON™ T500 compared to Fludora® Fusion though the difference was not statistically different (1.1% vs. 0.9%, p = 0.746). A similar trend was observed with EIR. EIR post-IRS was also lower with VECTRON™ T500 compared to Fludora® Fusion though the difference was not statistically significant.

**Table 3:** Sporozoite and entomological inoculation rates in *An. gambiae* s.l. collections from VECTRON™ T500 and Fludora® Fusion study arms.

	Before IRS		After IRS	
	Fludora fusion	VECTRON T500	Fludora fusion	VECTRON T500
N analysed	1282	1581	1094	874
N positive	24	32	12	8
Sporozoite rate (SR)	1.9	2	1.1	0.9
Mean cluster level sporozoite rate	2.2	3.6	1.2	1.4
Difference (95% CI)	1 (ref)	1.4 (− 0.9, 3.8)	1 (ref)	0.2 (− 1.1, 1.4)
P-value	ref	0.198	ref	0.746
Human biting rate (HBR)	2.30	2.77	0.77	0.67
Entomological inoculation rate (EIR)	0.043	0.056	0.008	0.006
Mean cluster level EIR	0.06	0.05	0.009	0.007
Difference (95% CI)	1 (ref)	− 0.01 (− 0.05, 0.03)	1 (ref)	− 0.002 (− 0.008, 0.004)
P-value	ref	0.444	ref	0.589

*HBR* mean bites per person per night, *EIR* infective bites per person per night.

### Insecticide susceptibility

**Figure 6:**
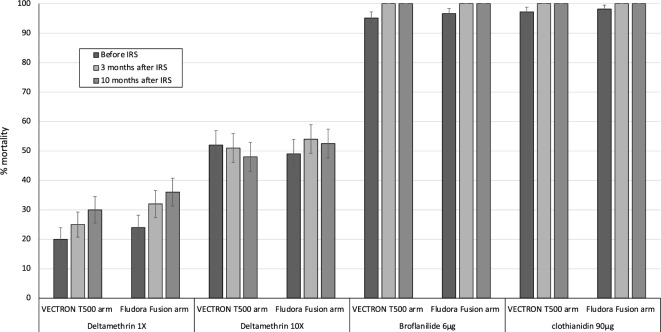
Mortality of wild *An. gambiae* s.l. from villages in VECTRON™ T500 and Fludora® Fusion arms in deltamethrin, broflanilide and clothianidin treated bottles at baseline and at 3 months and 10 months post-IRS. *A total of* ~ *400 adult mosquitoes collected as larvae from 4 villages in each study arm were tested at each timepoint.*

In total, 8921 mosquitoes from the study area were exposed to deltamethrin (12.5 µg/bottle and 120 µg/bottle), broflanilide (6 µg/bottle) and clothianidin (90 µg/bottle) in susceptibility bottle bioassays performed at baseline and at 3- and 10-months post IRS intervention. Mortality with deltamethrin was < 40% after exposure to the diagnostic dose of 12.5 µg/bottle and did not exceed 60% at the 10 × dose of 125 µg/bottle, indicating a high intensity of pyrethroid resistance in the study area (Fig. 6). With both broflanilide and clothianidin, mortality was > 95% at all timepoints indicating susceptibility to both insecticides and no change in susceptibility after the IRS applications (Fig. 6). No mortality was recorded in the untreated control bottles.

### Insecticide resistance genotypes

Table 4 presents the frequencies of the L1014F *Kdr* and G119S *Ace-1* mutations in a sub-sample of *An. gambiae* s.l. collected by HLC in the study area over the trial duration. The frequency of L1014F *Kdr* in the *An. gambiae* complex was generally high (> 80%) both before and after the IRS in both VECTRON™ T500 and Fludora® Fusion arms. These results indicated no increase in the frequency of L1014F *Kdr* after IRS application using either VECTRON™ T500 or Fludora® Fusion IRS. Regarding the G119S *Ace-1* mutation, the frequency was generally lower in *An. gambiae* s.l. across the study area (< 40%) and appeared to decline following IRS implementation. G119S *Ace-1* frequency was 15–36% before IRS in both study arms against 10–11% after IRS.

**Table 4:** L1014F (*Kdr*) and G119S (*Ace-1r*) gene frequencies in each study arm before and after IRS intervention.

	VECTRON T500		Fludora fusion	
Genotype	Before IRS	After IRS	Before IRS	After IRS
L1014F (*Kdr*)				
RR	174	158	173	152
RS	40	63	37	45
SS	20	6	16	18
Total tested	234	227	226	215
* Kdr* frequency	83%	83%	85%	81%
G119S (*Ace-1r*)				
RR	14	0	0	0
RS	145	46	67	44
SS	78	181	163	160
Total tested	237	227	230	204
* Ace 1r* frequency	36%	10%	15%	11%

### Residual efficacy (WHO wall cone bioassay results)

**Figure 7:**
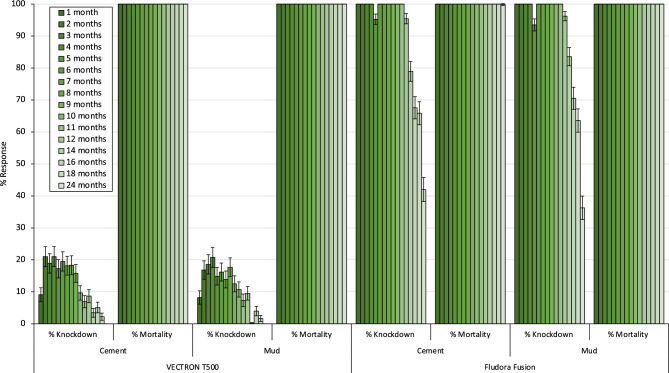
Knockdown (%) and mortality (%) of susceptible *Anopheles gambiae* s.s. Kisumu in monthly cone bioassays on IRS-treated mud and cement plastered house walls in VECTRON™ T500 and Fludora® Fusion arms.

**Figure 8:**
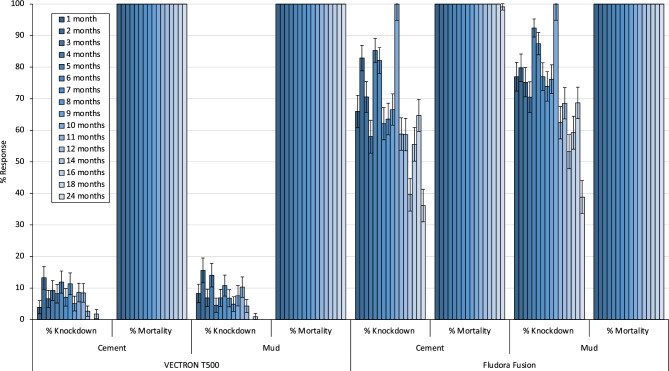
Knockdown (%) and mortality (%) of pyrethroid resistant *Anopheles gambiae* s.l. Covè in monthly cone bioassays on IRS-treated mud and cement plastered house walls in VECTRON™ T500 and Fludora® Fusion arms.

Mortality of the susceptible *An. gambiae* s.s. Kisumu and pyrethroid-resistant *An. gambiae* s.l. Covè strains was 100% in WHO wall cone bioassays performed on both cement and mud walled houses for all 18 months post-IRS in all the evaluation clusters in both study arms (Figs. 7 and 8). There was therefore no evidence of a decline in IRS residual activity for both insecticides over the 24 months of testing. Mosquito knockdown was higher with Fludora® Fusion compared to VECTRON™ T500 but this was expected given the rapid knockdown effect of the deltamethrin component in Fludora® Fusion. Mortality in the controls was < 5% at all timepoints.

### IRS spray quality results

**Figure 9:**
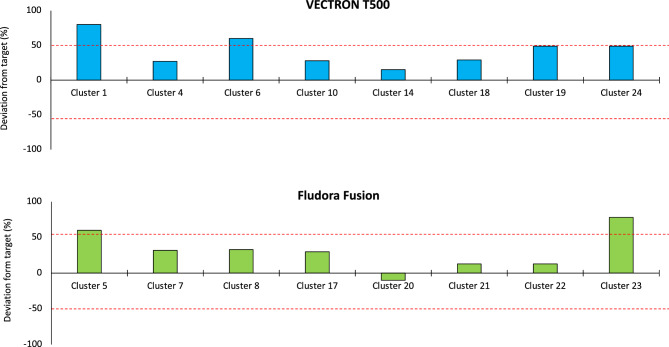
Deviation from target insecticide application rate based on number sachets used in each study cluster in VECTRON™ T500 and Fludora® Fusion study arms.

A total of 320 filter papers were analysed (160 from the Vectron™ T500 arm and 160 from the Fludora® Fusion arm) for chemical content to assess IRS spray quality. The filter paper chemical analysis showed an average active ingredient concentration of 214 mg/m^2^ (range 96–274) for broflanilide in VECTRON™ T500 clusters and 357 mg/m^2^ (range 107–661) for clothianidin and 45 mg/m^2^ (range 13–83) for deltamethrin in Fludora® Fusion clusters. These results showed an overall deviation from target application rate of 114% for VECTRON™ T500 and 78% for Fludora® Fusion. By contrast, insecticide application quantities based on the actual number of sachets applied in relation to the number required for the number of sprayable structures found in each cluster was within 50% deviation from the target rate in 80% of the study clusters (Fig. 9). Based on actual insecticide quantities applied, the spray applications were within an overall acceptable deviation from target of 31% for the Fludora® Fusion arm and 20% for the VECTRON™ T500 arm.

### Adverse events and acceptability

**Figure 10:**
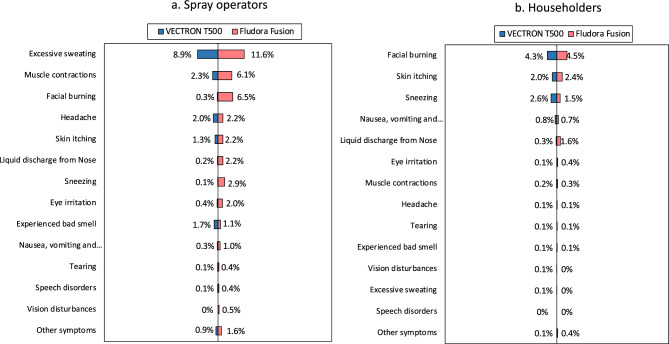
Proportion of adverse events reported in VECTRON™ T500 and Fludora® Fusion arms by spray operators and householders.

The summary results of adverse events reported by inhabitants of sprayed households and spray operators are presented in Fig. 10. Over the 22 days of the IRS campaign, spray operators reported fewer adverse events with VECTRON™ T500 arm compared to Fludora® Fusion arm (Fig. 10). Most adverse events were similar between both groups except for facial burning that was reported by more spray operators in the Fludora® Fusion arm (6%) compared to the VECTRON™ T500 arm. In a follow-up survey conducted 1 month after the IRS campaign, the spray operators did not report any adverse events. The number of adverse events reported from householders were higher at 1 week post spray (34/419 from the VECTRON™ T500 arm and 29/283 in Fludora® Fusion arm) and reduced substantially in both arms at 1-month post-spray. There were no major differences in proportion of adverse events reported by householders in the two study arms.

The main factors that influenced the acceptance of spraying were generally the same at surveys conducted immediately after the IRS and 6 months after irrespective of the study arms. Overall, the desire to reduce the mosquito population and other pests such as bedbugs, lice, flies, cockroaches, etc. in homes were the most frequent reasons put forward by the study participants. A small proportion of study participants (14%) from both study arms were concerned for the health of children and adults sleeping in sprayed homes in the survey conducted immediately after IRS, but these concerns were no longer raised at 6 months post-spray. Nevertheless, the difficulties in removing personal belongings and disruption of daily activities on the day of spray operations were raised at both acceptability surveys (just after IRS and 6 months later) in both study arms. Feedback received from heads of household about their perceptions of the effectiveness of the IRS products remained very positive at 6 months post-intervention survey for both study arms; over 90% reported that the IRS intervention was either excellent or good for them.

## Discussion

Whilst randomised controlled trials with epidemiological endpoints are the gold standard evidence for demonstrating the public health value of new vector control interventions^29^, they are expensive and unsuitable for some interventions. Because VECTRON™ T500 has already demonstrated non-inferiority to a WHO pre-qualified IRS insecticide in experimental hut studies^19,30^, according to WHO guidelines^31,32^, the insecticide can be covered by existing WHO policy recommendations for IRS without the need for more expensive randomised controlled trials with epidemiological endpoints. The current trial was, therefore, designed in line with existing WHO phase III protocols^33^ to build further evidence of the non-inferiority of VECTRON™ T500 to a WHO prequalified IRS insecticide in reducing malaria vector densities under large scale community use. Its entomological impact, residual efficacy, acceptability, safety, and operational feasibility was compared to Fludora® Fusion in communities in the Za-Kpota District of Benin where the vector population exhibits a high intensity of resistance to pyrethroids but is susceptible to clothianidin and broflanilide.

The results from this community trial demonstrate compelling evidence of a comparable impact of VECTRON™ T500 to Fludora® Fusion in reducing the risk of malaria transmission at a community level when deployed for IRS on a large scale against a background of high coverage with pyrethroid-only ITNs. VECTRON™ T500 was non-inferior to Fludora® Fusion in reducing the human biting rate of *An. gambiae* s.l. mosquitoes in HLC collections both indoors and outdoors and showed lower sporozoite rates and entomological inoculation rates compared to Fludora® Fusion, although the latter were not statistically significant. Vector biting in clusters receiving VECTRON™ T500 was generally comparable to Fludora® Fusion at all HLC timepoints for up to 18 months post-IRS deployment. The insecticide also showed high acceptability with householders with very few perceived adverse events reported. These findings confirm the community impact of VECTRON™ T500 for IRS, demonstrating that its recent addition to the WHO list of prequalified products^21^ presents a new, effective IRS mode of action for malaria control at the community level.

The main reason put forward for the declining use of IRS for malaria control is the high costs and logistical constraints associated with sustaining annual IRS campaigns in high endemic settings^8,9^. IRS formulations that offer prolonged efficacy, thus requiring less frequent campaigns, are more desirable^34^ as they are potentially more cost effective than shorter-lived IRS insecticides. The residual efficacy of an IRS formulation is usually indicated by the number of months for which mosquito mortality in WHO cone bioassays on IRS treated home walls remains ≥ 80%^33^. In this study, VECTRON™ T500 induced 100% mortality in in situ wall cone bioassays for 24 months post-IRS application on cement and mud treated household walls with both susceptible and pyrethroid-resistant strains of *An. gambiae* s.l. demonstrating remarkable residual efficacy. Our study tested the residual activity of Fludora® Fusion beyond 12 months^35^ for the first time and further demonstrated a prolonged cone bioassay activity with the insecticide also lasting up to 24 months post-IRS implementation. The findings with VECTRON™ T500 corroborate previous studies that showed over 18 months of residual activity of VECTRON™ T500 on IRS treated mud and cement walls in experimental hut studies in Benin^19^. Based on this long residual efficacy, VECTRON™ T500 may be effective in reducing malaria transmission for much longer periods compared to other IRS formulations of other insecticides, thus requiring less frequent IRS campaigns (e.g., every 2 years) and providing a more cost-effective option.

The development of vector resistance to public health insecticides is recognised as a major threat to progress in achieving malaria elimination targets^14^. Efforts must, therefore, be made to preserve the efficacy of new malaria vector control insecticides as they become available. The rotational deployment of IRS insecticides with different modes of action has the potential to reduce selection pressure for insecticide resistance genes in malaria vector populations and is thus recommended for pre-emptive management of insecticide resistance^15^. However, this strategy has not been adequately explored for malaria vector control due to the limited choice of insecticide modes of action that were available for IRS^36^. The addition of broflanilide (VECTRON™ T500) to the WHO list of prequalified IRS products—a new mode of action with no observed cross resistance to other public health insecticides—will substantially improve capacity to manage insecticide resistance and preserve the efficacy of newly developed IRS insecticides using the IRS rotation strategy.

Given the goal of universal ITN coverage implemented by most national malaria control programmes, most IRS campaigns are deployed against a background of medium to high coverage with ITNs. While current WHO guidance recommends against a combined ITN and IRS approach^37^, considering the high persistence of malaria despite high coverage with ITNs in many hyperendemic countries^14^ and the poor durability and retention rates of ITNs by users^38^, it will be very challenging to improve the stalling progress against malaria and achieve elimination with ITNs alone. Deploying a long-lasting IRS like VECTRON™ T500 to complement novel ITNs later in their operational life in high transmission areas might be an effective way to further reduce malaria transmission while reducing costs associated with sustaining annual IRS campaigns. Our previous studies have, however, demonstrated that the impact of such combinations would depend on the interactions that exist between the IRS insecticide and the insecticide used on the ITNs^39–42^. To help guide the deployment of VECTRON™ T500, further studies to understand its impact when combined with different types of ITNs currently available for malaria control are advisable. Co-deployment of VECTRON™ T500 with new dual active ingredient ITNs may also provide opportunity to manage insecticide resistance by presenting two different modes of action to the vector population at the same time^15^.

## Conclusion

VECTRON™ T500 was non-inferior to Fludora® Fusion in reducing vector densities and malaria transmission by pyrethroid resistant vectors when applied for IRS in communities in central Benin. The insecticide showed prolonged efficacy on home walls lasting up to 24 months and a high acceptability to homeowners. Community application of VECTRON™ T500 for IRS provides improved and prolonged control of pyrethroid resistant malaria vectors and enhances capacity to manage insecticide resistance.

## Data Availability

The datasets used and/or analysed during the current study available from the corresponding author on reasonable request.

## Abbreviations

PBO: Piperonyl butoxide
ITN: Insecticide treated nets
WHO: World Health Organization
PQT: Prequalification team
AI: Active ingredient
GLP: Good laboratory practice
CREC: Centre de recherche entomologique de cotonou
LSHTM: London school of hygiene and tropical medicine
PAMVERC: Pan African Malaria Vector Research Consortium
